# Primary Non-Adherence to Antihistamines—Conclusions From E-Prescription Pilot Data in Poland

**DOI:** 10.3389/fphar.2020.00783

**Published:** 2020-05-21

**Authors:** Grzegorz Kardas, Michał Panek, Piotr Kuna, Janusz Cieszyński, Przemysław Kardas

**Affiliations:** ^1^Clinic of Internal Diseases, Asthma and Allergy, Medical University of Lodz, Łódź, Poland; ^2^Ministry of Health, Warsaw, Poland; ^3^First Department of Family Medicine, Medical University of Lodz, Łódź, Poland

**Keywords:** antihistamines, antihistamine drugs, non-adherence, primary non-adherence, e-prescription

## Abstract

**Background:**

In allergic conditions such as allergic rhinitis and urticaria, orally administered H_1_-antihistamines belong to first-line therapy and therefore, are widely prescribed. Due to the frequent, and often chronic, course of allergic diseases, adherence is of great importance. In 2018 a novel, nationwide e-prescription system was piloted in Poland, which allowed to analyze primary non-adherence to orally administered H_1_ antihistamines.

**Objectives:**

To assess the primary non-adherence to orally administered H_1_-antihistamines in Poland, defined as not redeeming the drug issued on a particular e-prescription within its validity period.

**Methods:**

The study was based on all e-prescriptions issued in Poland in 2018, issued for 119.880 drugs. The analysis included nine major orally administered H_1_ antihistamines available in Poland.

**Results:**

Out of 2280 analyzed e-prescriptions on orally administered antihistamines, 1803 (79.1%) of them were redeemed. Therefore, the level of primary non-adherence reached 21%. Among women it reached 19.9%, but it was not significantly lower than among men (23.4%, p=0.064). The highest non-adherence (31.3%) was observed in the age group 19-39, whilst the highest adherence rate (84.6%) was observed in those 75 years or older. The most frequently prescribed second-generation antihistamine was bilastine—596 e-prescriptions with 23.7% primary non-adherence.

**Conclusions:**

More than 1 out of 5 e-prescriptions on orally administered H_1_-antihistamines were not redeemed in Poland in 2018. Age, but not gender, significantly influenced the degree of primary non-adherence to these drugs. To authors knowledge, this is the first real-life study on primary non-adherence to H_1_-antihistamines in Poland and one of the very few on this subject worldwide.

## Introduction

Adherence to recommended treatment is one of the fundamental factors affecting the efficacy of treatment across medical fields. In general, the worse the adherence, the worse the health outcomes, and patients quality of life (Kleinsinger, 2018). Moreover, adherence is also one of the major determinants of the health care costs (Iuga and McGuire, 2014). This is no different in case of medicines used in management of allergic diseases, e.g. allergic rhinitis (AR) or urticaria. In course of these diseases, the use of H_1_-antihistamines (addressed as ‘antihistamines' throughout the whole text) is particularly important, with an emphasis on second generation drugs.

Allergic rhinitis (AR) is a common chronic respiratory disorder affecting upper airways. Its clinical manifestations vary worldwide due to diversity of the climate and the associated different course of weather seasons. Symptoms of AR include nasal itching, sneezing, rhinorrhoea, and nasal congestion, frequently accompanied by ocular symptoms—itching and redness of the eyes and tearing (Brożek et al., 2017). Although the disease is not life-threatening, the associated troublesome symptoms adversely affect patients quality of life and their work effectiveness (Bousquet et al., 2013). Epidemiological data indicates that self-reported AR presently affects up to 40% of adults and 2% to 25% children worldwide (Asher et al., 2006; Brożek et al., 2017). Studies show that around 23% of the population is affected by AR in Europe (Bauchau and Durham, 2004) and 12% to 30% in the United States (Nathan et al., 2008). Frequent occurrence of this disease has also been demonstrated in other regions of the world: 7% to 54% in Africa, 7% to 45% in Middle East, 12% to 41% in Australia, 1.6% to 43% in China/Taiwan, and 12% to 41% in Australia (Katelaris et al., 2012). What is important, a clear upward trend in the incidence of the disease was observed in Western European countries. It has been shown, for example, that in years 1991 to 2010 in Italy the prevalence of AR increased from 16.8% to 25.8% (de Marco et al., 2012). An increase in the prevalence of AR and urticaria has also been demonstrated in some dynamically economically and industrially developing countries in Asia (Kulthanan et al., 2018). This data clearly indicates frequent prevalence of this disease worldwide and its significant impact on health of millions of people. Thus, the global cost of treating AR is substantial. In particular, it is dependent on disease severity and comorbidities. In the United States, the overall costs of treating AR in 2005 was estimated at US $11.2 billion, with the total direct medical cost—prescription medication and outpatient visits—reaching $3.4 billion (Meltzer and Bukstein, 2011).

Another important and highly prevalent allergic disease is urticaria. It is characterized by development of wheals (hives), angioedema or both. The overall prevalence of acute urticaria is estimated to reach 20% of patients (Zuberbier et al., 2018). In a recently published, representative questionnaire based study on epidemiology of urticaria in Poland, 11.2% of respondents have experienced symptoms of urticaria at least once in their life. The majority—10.6% cases—were acute, while 0.6% study participants reported chronic urticaria (Raciborski et al., 2018). The disease has a significant impact on patients quality of life and work efficacy (Rutkowski and Grattan, 2017). A cross-sectional study of 176,000 adults from five European Union countries (France, Germany, Italy, United Kingdom, and Spain) revealed a 0.5% point prevalence of CU in adults and US $0.2 receiving prescription medication. Among these patients, CU resulted in decreased work and private life QoL and numerous health-related impairments, including self-reported depression, anxiety and sleep disorders caused by CU compared with healthy controls (Balp et al., 2015).

In allergic conditions, such as AR and urticaria, antihistamines belong to evidence-based first-line therapy, and are, therefore, widely prescribed (Antia et al., 2018; Zuberbier et al., 2018; Emeryk et al., 2019; Bousquet et al., 2020). Currently, numerous drugs from this group are available worldwide, of which the most widely used are currently second-generation antihistamines, e.g., bilastine, loratadine, desloratadine, cetyrisine, levocetirisine etc. In Poland, some of these drugs (e.g., cetirizine, fexofenadine, loratadine) are currently available over the counter. However, most of the antihistamines are still available on prescription only. This is mainly related to their profile of side-effects, which include sedation (mainly in case of the first-generation antihistamines).

Due to the frequent, and often chronic, course of allergic diseases, the key to managing them is the regular use of drugs, including antihistamines. The selection of a specific medication depends on the indication, the previous response to treatment, severity of the disease, and patient preferences (Kuna et al., 2016). Out of the antihistamines available on the market, various substances have proven efficacy and safety (Horak et al., 2010; Sánchez-Borges and Ansotegui, 2019). However, comparative studies of their efficacy are limited (Church and Maurer, 2012). The newest EAACI/GA²LEN/EDF/WAO guidelines highlight the importance of long-term antihistamine treatment in CU. The algorithm of CU treatment includes second-generation antihistamines as first-line treatment, but it is not distinguished which of the drugs is the most effective (Zuberbier et al., 2018).

The symptoms of allergic diseases vary among patients and may vary over time, both in type and severity. Being predominantly not life-threatening conditions, they leave the patients much more freedom in self-managing the treatment then other prevalent conditions, such as e.g. hypertension. Nevertheless, in long-term allergy management the proper use of a drugs is an essential determinant of the therapeutic success. Therefore, assuring adherence to medication is a need.

According to ABC European consensus, medication adherence has been defined as an active, cooperative, and voluntary participation of the patient in following recommendations from a health care provider, involving three critical steps:

- Initiation, which defines the moment that the patient takes the first dose;
- Implementation, which is related with the extent to which the prescription regimen was followed;
- Discontinuation, which happens when the patient stops taking the prescribed medicines (Vrijens et al., 2012).

Various methods of adherence measurement allow for assessment of different elements of this taxonomy. For example, comparing prescribing and dispensing data allows for detection and measurement of primary non-adherence. Pharmacy claims databases' analyses allow for longitudinal assessment of implementation, and detection of discontinuation, yet are not sensitive to identify daily variation of implementation. Electronic monitoring is a gold standard for assessment of details of drug taking histories, yet is too obtrusive and costly to be routinely implemented in a daily practice. Thus, none of available methods could be accepted as universal solution for every possible settings (Vrijens et al., 2012). Assuming that prescription for a drug is a proof that at the moment, medical professional assessed the need for pharmacotherapy, primary non-adherence, defined as not obtaining a prescribed medication from the pharmacy, is a serious deviation from agreed treatment plan. The prevalence of primary non-adherence has been an issue of many studies, especially in management of chronic non-communicable diseases—such as hypertension, diabetes, and other (Raebel et al., 2012; Fallis et al., 2013; Pottegård et al., 2014; Aznar-Lou et al., 2017). However, only limited data are available on this issue regarding the treatment of allergic conditions. In an ongoing, long-lasting therapy, the key to clinical success is also secondary adherence, defined as receiving dispensings or refills as prescribed during a defined observation period (usually 6–12 months) (Raebel et al., 2013). So far, primary non-adherence in general, and particularly for antihistamines, has not been extensively studied in Poland due to lack of reliable data. However, the recently implemented, nationwide eHealth system in Poland, created a new opportunity for that type of studies, enabling comparisons of data between pharmacy claims and prescriptions. With many other major advantages over traditional prescriptions, e-prescription system allows to trace primary non-adherence through the comparison of data regarding prescribed and dispensed drugs.

### Aim of Study

The aim of this study was to analyze the extent of primary non-adherence to selected orally administered antihistamine medications available in Poland with indication of treatment of AR and urticaria overall and for individual drugs. Also, we wanted to find out whether patient demographics had an effect on this phenomenon.

## Methodology

In this retrospective analysis, we used data from all (119,880) e-prescriptions issued in Poland in 2018 as part of a national e-prescription pilot, which included 43 medical centers (primary care, outpatient specialist clinics, and hospitals in 9 of 16 districts of Poland). Participating health care institutions were either invited by CSIOZ (see below), or joined on the voluntary basis. The prescriptions were issued by 190 medical doctors, from various fields of medicine.

The data used in this study were provided by the Center of Information Systems for Healthcare (Centrum Systemów Informacyjnych Ochrony Zdrowia, CSIOZ), which is a governmental institution responsible for the digitalization of the Polish health care system. One of its recent tasks is to implement the now fully-operational, nationwide system of e-prescriptions.

The database was fully anonymized; therefore, according to the policy of Ethical Commission of Medical University of Lodz, the study was not subject to ethical approval. It was also simplified: along with basic characteristics of the patient (namely, age and sex), each record included the date of prescription, details (such as drug trade name, dose, number of packs) of the prescribed drug, date of dispensation (if it took place), and details of the drug being dispensed.

Primary non-adherence is often defined as failure to pick up a medication within a defined number of days after the prescription was made (Raebel et al., 2013). The data presented in this paper, however, did not include clinical data, nor were the longitudinal prescription histories for individual patients available. Therefore, for the purpose of this study, primary non-adherence was defined as not having dispensed an individual e-prescription within 30 days, which is the typical period of prescription validity in Poland.

In our analysis, we included all orally administered antihistamines available in Poland, with all of their available doses and formulations. The inclusion criteria for a particular substance to be analyzed in the study were: (1) available oral formulation (pill, tablet, drops etc.) and (2) >10 e-prescriptions issued. Thus, the final analysis consisted of nine compounds in total—full list is available in **Table 1**.

**Table 1:** List of the analyzed compounds with their ATC codes.

No.	ATC CODE AND NAME
1.	N05BB01 Hydroxyzine
2.	R06AE07 Cetirizine
3.	R06AE09 Levocetirizine
4.	R06AX13 Loratadine
5.	R06AX17 Ketotifen
6.	R06AX26 Feksofenadine
7.	R06AX27 Desloratadine
8.	R06AX28 Rupatadine
9.	R06AX29 Bilastine

Data analysis included descriptive statistics of overall prevalence of primary non-adherence. Then, the effect of potential drivers of primary non-adherence (age and gender) was assessed. For the purpose of this analysis, age (a continuous variable) was categorized into 5 groups: 1 to 18 years, 19 to 39 years, 40 to 64 years, 65 to 74 years, and 75 years or older. Categorical variables were expressed as proportions and compared between relevant groups using the χ^2^ test. For statistical calculations, Statistica 10 software (TIBCO Software Inc.) was used. A *P* value of less than 0.05 was considered significant.

## Results

Of 119,880 individual drugs prescribed in Poland on e-prescriptions, 2,280 (1.9%) were orally administered antihistamines of interest for this study (17 e-prescriptions did not meet the inclusion criteria). Of these, 1,803 (79.08%) of e-prescriptions were redeemed, thus the level of primary non-adherence for these antihistamines reached 20.98%. For second-generation antihistamines only, the level of primary non-adherence was 22.82%.

The possible differences in primary non-adherence to orally administered antihistamines among genders were analyzed. Out of all e-prescriptions on these drugs, 1,608 (70.5%) were prescribed for women. Primary non-adherence in this group reached 19.9%, but was not significantly lower than among men (23.4%, p=0.064)—detailed information is available in **Table 2**.

**Table 2:** Primary non-adherence to antihistamines by gender, chi^2^ = 3,435; p = 0.064.

Patient	Gender				Summary	
	Male		Female			
	n	%	n	%	n	%
**Adherent**	515	76.6	1,288	80.1	1,803	79.1
**Non-adherent**	157	23.4	320	19.9	477	20.9
**Summary**	672	100.0	1,608	100.0	2,280	100.0

Statistically significant difference in the mean age of adherent vs. non-adherent patients was observed. It turned out that the patients who filled the e-prescriptions were significantly older on average than those who did not (58.9 ± 19.8 years vs. 53.6 ± 19.8 years, respectively, *p* = 0.000).

**Figure 1:**
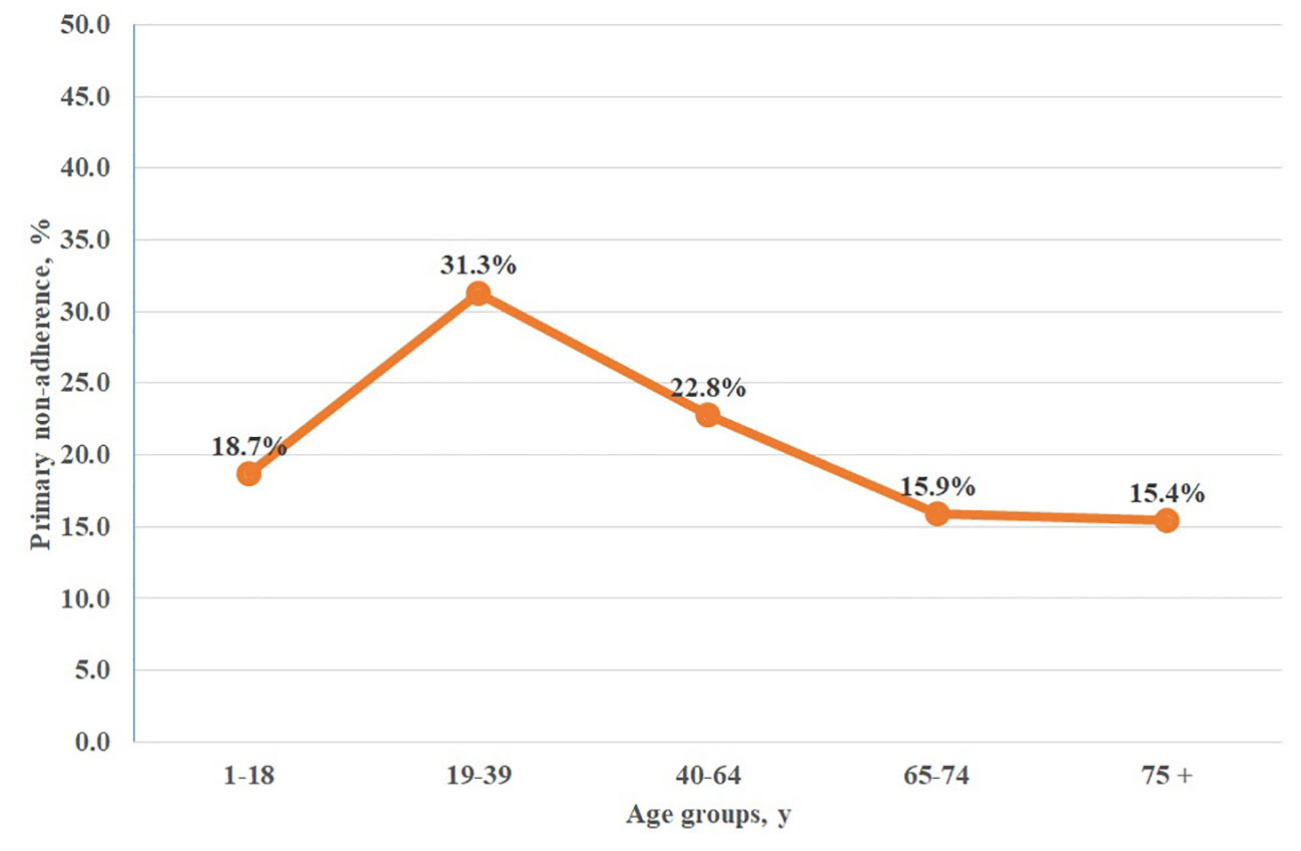
Primary non-adherence to orally administered antihistamines among age groups in Poland. chi^2^ = 41,810; p = 0.0000.

Further analysis of age-related primary non-adherence to antihistamines revealed significant differences across age groups. The highest non-adherence (31.3%) was observed in the age group 19 to 39 years, while the highest adherence (84.6%) rate was observed in 75 years or older group (**Figure 1**).

Among the analyzed drugs, the most numerous e-prescriptions (781) were issued for first-generation antihistamine—hydroxyzine. Primary non-adherence to this drug was 17.29%. The most frequently prescribed second-generation antihistamine was bilastine—596 e-prescriptions with 23.66% primary non-adherence. The least frequently prescribed antihistamine was ketotifen, with only 14 e-prescriptions and 28.57% primary non-adherence. With 101 e-prescriptions, the antihistamine with the highest primary non-adherence was loratadine (101 e-prescriptions and 33.66% non-adherence). The drug with second highest non-adherence was levocetirizine—152 e-prescriptions and 32.89% primary non-adherence (**Table 2**).

## Discussion

Oral antihistamines are considered effective in reducing the major symptoms of allergic rhinitis, urticaria and other allergic diseases. They are recommended at all steps of AR and urticaria therapy. Since these drugs are generally administered as a single daily dose, they seem to create good grounds for patient adherence. Nevertheless, successful management of particular allergic diseases undoubtedly depends on patient adherence with the recommended treatment. This, in turn, is affected by various factors: socio-economic (e.g., family support, employment status) health care team and system-related (e.g., proper information on drug administration), condition-related (e.g., presence or lack of present symptoms), drug-related (e.g., dosing regimen, formulation, cost of the drug), and patient-related (e.g., education, psychological profile, health beliefs, cognitive functions), etc. (Kardas et al., 2013). For comparison, it is worth noting that the other options of allergy treatment, in particular AR treatment—topical antihistamines, topical cromones, immunotherapy—are considered to achieve lower adherence due to the more frequent dosing schedule and poorer efficacy in managing currently occurring symptoms (van Cauwenberge, 2005).

This study presents a number of interesting results regarding primary non-adherence to medication in allergic diseases. With the use of high-quality, unbiased data available from a nationwide e-prescription database, we have demonstrated a substantial level of primary non-adherence to orally administered antihistamines. The results of the study show that age, but not gender impact the adherence levels to these drugs, which is an important clinical information (see **Table 2** and **Figure 1** for detailed information). The non-adherence to this particular drug group matched that obtained in our previous study on drivers of non-adherence in Poland performed with the same database, where non-adherence to drugs in six major areas (diabetes, antithrombotic agents, cardiovascular system, lipid modifying agents, antibiotics, and psychoanaleptics) reached 20.8% (Kardas et al., 2020). Of big importance is the fact that this study is the first to assess the primary non-adherence to orally administered antihistamines in Poland and one of the very first of such studies worldwide. Moreover, the methodology used in this study allowed for objective assessment of non-adherence also for particular drugs (**Table 3**). Other methods of adherence assessment, such as questionnaires, diaries, indirect assessment by practitioners, or even pill count, are subject to large bias, and in general, tend to underestimate the levels of non-adherence (Basu et al., 2019). Still, in the available literature we can find some examples confirming the observed phenomenon.

**Table 3:** Primary non-adherence to individual antihistamines.

ATC code, drug name	E-prescriptions issued	E-prescriptions redeemed	Primary Adherence	Primary Non-Adherence
**N05BB01 Hydroxyzine**	781	646	82.71%	17.29%
**R06AE07 Cetirizine**	216	172	79.63%	20.37%
**R06AE09 Levocetirizine**	152	102	67.11%	32.89%
**R06AX13 Loratadine**	101	67	66.34%	33.66%
**R06AX17 Ketotifen**	14	10	71.43%	28.57%
**R06AX26 Fexofenadine**	145	122	84.14%	15.86%
**R06AX27 Desloratadine**	139	118	84.89%	15.11%
**R06AX28 Rupatadine**	136	111	81.62%	18.38%
**R06AX29 Bilastine**	596	455	76.34%	23.66%
**Total**	**2280**	**1803**	**81.58%**	**18.42%**

A study based on a similar approach that was used in this study was published by Fischer et al. In this paper, authors analyzed primary non-adherence to various medications using data from 195,930 e-prescriptions issued in the United States. In that study that primary non-adherence to “allergy medications” reached 28.7%. For newly prescribed drugs from that field the percentage of e-prescriptions not filled in reached 30.9% (19). In this study, the authors also analyzed a population narrowed down to patients using the e-prescription solution. Another study, also by Fischer et al. using similar methodology revealed 30.0% of primary non-adherence to “cold/cough/allergy” medications (Fischer et al., 2010; Fischer et al., 2011).

In a study by Koberlein et al., the overall non-adherence to desloratadin administered because of allergic rhinitis in patients aged 11 to 101 years was only 1.9%. Although the cohort of this study was large (42,111 patients), the results of this study should be read carefully due to the methodology used. Namely, adherence was assessed by managing doctors on a subjective scale “excellent”/”good”/”moderate”/”poor” over a mean treatment duration of 41.6 days, which does not allow for a fully objective analysis of the discussed phenomenon. Despite this, this study has identified several factors affecting antihistamine adherence—concomitant disease, especially psychiatric disorder or asthma, increased the chances for patient non-adherence (Köberlein et al., 2013).

In another study, which was a multicenter, open-label, phase IV study of rupatadine, adherence of 324 patients with allergic rhinitis was measured by means of a pill count at each monitoring visit. In treatment period of 1 to 6 months adherence level to rupatadine reached 89.6%,while for 1 to 12 months treatment it was 83.26%. Although this study was conducted using a more reliable methodology, it is worth noting that a significant impact on adherence could have had the setting of a clinical study and associated frequent monitoring visits (Valero et al., 2009). Possible bias to the studies based on that methodology might be brought by the fact that sometimes patients forget to bring, or purposefully discard unused drugs.

Among 751 patients with chronic spontaneous urticaria Keneko et al. studied adherence to treatment and found low adherence to oral treatment (65.4% of patients) using the eight-item Morisky Medication Adherence Scale questionnaire (Kaneko et al., 2015). Interestingly, another study by Heng et al. revealed that low medication adherence in patients with chronic urticaria was not dependent on the dosing frequency scheme (once daily dosing versus more frequent ones) (Heng et al., 2015).

Unfortunately, there are no studies available to compare the levels of primary and secondary adherence in Poland. On the other hand, it has been found that adherence to long-term treatment was poorer in Poland than in West European countries—e.g. in a large international study, non-adherence to antihypertensives reached 44% across studied European countries, whereas for Poland it was 58% (Morrison et al., 2015). In general, multiple antihistamines may be used interchangeably, due to very similar profile of their activity. Therefore, various level of primary adherence might be an important suggestion for clinicians which one to prioritize when prescribing, in order to increase primary adherence, and help obtaining best health outcomes. Individual properties of the compound (e.g., effectiveness, adverse effects profile) and particular product (e.g., drug formulation such as capsules vs tablets, tablet size, color, and ease of swallowing, out-of-pocket cost, etc.) are affecting patient decisions—which may contribute to the difference adherence to various compounds (**Table 3**). Recent study in Polish outpatients found out that patient priorities regarding various attributes of oral solid drug forms vary significantly between short-term and long-term treatments, and patients are happy to pay significantly more for drugs of their preferred characteristics (Kurczewska-michalak et al., 2020).

A certain limitation of our study was that due to the methodology we used in it, in particular the structure of the analyzed database, it was not possible to determine what were the exact clinical reasons for a particular e-prescription nor to determine the adherence level for a particular disease. The database was anonymous and did not include clinical data, namely the diagnosis and did not allow to analyze the secondary adherence. However, the presented data may still be considered as an objective picture of adherence in the area of allergic diseases treated with oral antihistamines. It is also important to note that due to the pilot nature of the new health care system tool in Poland, an e-prescription, this fact could have had an impact on primary non-adherence. Nevertheless, data obtained with this method can be considered as high quality and unbiased because it is not self-reported by patients nor subject to physicians' opinion on patients adherence. Moreover, it is worth noticing that the lowest level of primary non-adherence was observed in the 75 years or older group, which does not confirm a technological barrier toward using this solution by the eldest seniors. The analyzed data has been obtained from a nationwide database, which included all the e-prescriptions issued in the analyzed period, which allows to consider it complete. To the authors' knowledge, this is the first study of primary non-adherence to antihistamine medications in Poland and one of the first to address the issue with the e-prescription data methodology worldwide. Further analyses of data continuously collected using the national e-prescription system compounds (electronic patient account, e-medical leave and other) may bring even more in-depth assessment of drivers of primary non-adherence to antihistamines in Poland. Nevertheless, even with these preliminary findings, we collected evidence that might be of high usefulness in health and medicine policies, and certainly, will be more than helpful in designing any future analyses.

Another limitation of the study was that we could only analyze primary non-adherence, meaning the analysis concerned on the action of redeeming or not redeeming a particular prescription. The information regarding the number of doses each patient took—or skipped—after filling a particular e-prescription was not possible to be measured. However, this was impossible to be analyzed within the database used and in fact was not within the objectives of this work.

Finally, one more limitation of this study is related to the fact that studying primary non-adherence with e-prescription data, we do not collect the information on the reasons for this behavior. These might have been diverse, from lack of trust in diagnosis or doctor, through high out-of-pocket costs of drugs (in Poland, patients pay various co-payments, which level varies according to the drug, indication, patient characteristics, etc), OTC availability of several antihistamines, up to drug formulation, etc. (Kardas et al., 2013).

Regardless of the reasons of the primary non-adherence to orally administered antihistamines—and other medications in general—some suggestions as to the corrective actions could be found in the literature. For example, a randomized trial among patients with AR revealed that a daily short message service reminder improves adherence to intranasal corticosteroid treatment (Wang et al., 2014). A similar solution—a SMS service among asthmatic patients reminding them daily to take their anti-asthmatic medication proved to be effective and increase adherence by 17.8% (Strandbygaard et al., 2010). Using the same approach, involving a reminder to redeem an e-prescription, could be beneficial in achieving better primary adherence. This could be simply achieved and implemented in the e-prescription solution, which in fact includes SMS service, by sending a reminder if the expiration date of a particular e-prescription would be approaching. Moreover, a similar approach may be implemented to inform the prescriber of non-adherence, e.g., *via* the e-prescription desktop application. Additionally, a feature that would highlight a not redeemed, but frequently prescribed drug would also be of high value. Altogether, with this e-health solution, we believe it creates a great opportunity for discussion with the patient on barriers towards adherence. We recommend that this shall be done with a pro-active dialogue technique, that is, not blaming the patient for being non-adherent, but encouraging a productive discussion on real-life barriers towards adherence. Bearing in mind the reported levels of primary non-adherence practitioners shall once again reflect that prescribing a drug is not equal to the act of redeeming it by the patient, and subsequently taking the medicine.

Another study aimed at improving adherence to inhaled corticosteroids (ICS) in asthma showed a modest but significant improvement among patients who received interactive voice recognition phone calls reminding them of medication refills and a need for continuous ICS treatment. This sort of solution, similar to SMS service, may be another option to improve primary adherence (Vollmer et al., 2011). Further approaches may also include usage of mobile apps to address the clinical significance of proper allergy diagnosis and further encourage adherence (Cingi et al., 2015; Bousquet et al., 2017).

In general, once acknowledging the results of the study, a reconsideration of prescribing antihistamines is strongly advised. As more than 1/5 of antihistamines in general, and almost 1/3 among young adults are not redeemed, this may reflect on the issue of over-prescription of these particular drug group. The levels of primary non-adherence report not only on patients' conscious decision on not to but a drug, but also of prescribing those drugs excessively by the practitioners themselves. The reasons for this observation and for reasons on the primary non-adherence to this drug group require further research.

## Conclusions

In this study, we have shown that more than one of five e-prescriptions on orally administered antihistamines were not redeemed in Poland in 2018. Age, but not gender, significantly influenced the degree of primary non-adherence to these drugs. Particular orally administered antihistamines varied in terms of primary non-adherence range between 15.11% for desloratadine and 33.66% for loratadine. To authors knowledge, this is the first real-life study on primary non-adherence to antihistamines in Poland and one of the very few on this subject worldwide, which make it useful in clinical practice for general practitioners, allergy specialists, dermatologists, pulmonologists and other specialists, and also for health-policy makers, payers, etc.

## Data Availability Statement

The datasets for this article are not publicly available because of market potential (records hold tradenames of the drugs prescribed). Requests to access the datasets should be directed to corresponding author, PKa.

## Author Contributions

GK, MP, PKu and PKa created the concept of the paper. JC organized and provided the database used in the study. GK, MP, PKu and PKa contributed to the analysis. GK drafted the paper, which was reviewed and approved by JC, MP, PKu and PKa.

## Funding

The study was funded by the Medical University of Lodz, Poland.

## Conflict of Interest

The authors declare that the research was conducted in the absence of any commercial or financial relationships that could be construed as a potential conflict of interest.
